# Uncovering the Hidden Threat: A Case Report of Suspected Dengue Fever in Armenia

**DOI:** 10.7759/cureus.40722

**Published:** 2023-06-21

**Authors:** Md Foorquan Hashmi, Fiza Khan, Gohar Matevosyan, Vigen Asoyan, Alvard Hovhannisyan

**Affiliations:** 1 Department of General Medicine, Yerevan State Medical University, Yerevan, ARM; 2 Department of Infectious Diseases, Yerevan State Medical University, Yerevan, ARM

**Keywords:** hemorrhagic fever of unknown cause, progressive leukopenia, thrombocytopenia, viral infection, aedes, dengue hemorrhagic fever (dhf), dengue

## Abstract

Dengue is a viral infection transmitted by mosquitoes that causes fever, headache, joint pain, nausea, vomiting, and pain behind the eyes. In severe cases, it can progress to dengue hemorrhagic fever and dengue shock syndrome, which can be life-threatening. Armenia has not reported a single case of dengue to date and is non-endemic for this disease. However, it has been found that the vector of the disease, *Aedes albopictus*, is present in Armenia since 2016. The aim of this report is to present the imported case of suspected dengue hemorrhagic fever.

A 23-year-old female who was admitted to the University Hospital experienced symptoms of general weakness, fever, joint pain, and chills after her return from Bali and had a three-day febrile period. A thorough examination revealed mosquito bites on her skin. On the fourth day of hospitalization, the patient's condition deteriorated. She started experiencing vaginal bleeding. On the same day, the patient noted a small petechiae rash sized 1-2 mm in diameter in the upper and lower extremities. The patient deteriorated, with progressive leukopenia and thrombocytopenia, and hypertransaminasemia. Screening tests for HIV and hepatitis A, B, C, and E were performed, and the results showed that the anti-hepatitis C antibody was positive, while the hepatitis C virus polymerase chain reaction was negative. The case was reported to the National Center for Disease Control and Prevention as an imported case of hemorrhagic fever. Unfortunately, no lab test was available there for confirmation of the diagnosis. The patient received IV infusion and symptomatic treatment. Her condition improved, and upon discharge, she was in a state of recovery.

This case report highlights the importance of early diagnosis and appropriate treatment for hemorrhagic fevers, particularly dengue fever. The unavailability of diagnostic kits for dengue in Armenia highlights the need to invest in improving their availability. It also emphasizes the importance of maintaining dengue surveillance in non-endemic nations and carefully evaluating and monitoring febrile patients who have returned from dengue-endemic countries.

## Introduction

Dengue is a virus transmitted by mosquitoes and is the leading cause of arthropod-borne viral diseases worldwide [1]. The virus is spread by several species of *Aedes* mosquitoes, primarily *Aedes aegypti*, *Ae. polynesiensis*, *Ae. scutellaris*, and *Ae. albopictus*. Dengue fever (DF) is caused by any of four distinct serotypes: DEN-1, DEN-2, DEN-3, and DEN-4 [1]. Dengue virus is a single-stranded RNA (ssRNA) virus with a positive-sense RNA (5’ to 3”) and belongs to the *Flaviviridae* family. The viral RNA can be directly translated into viral protein. Dengue disease can have a variety of severe and non-severe clinical symptoms, and symptoms usually begin three to four days after infection [2]. It often lasts between five and seven days, and it may also be biphasic, have a remittent pattern, or be a low grade [2]. Its clinical features are similar to those of other illnesses, which can result in incorrect diagnoses or delays in receiving appropriate treatment. Due to the severity of the muscular and joint pain and spasms, it is often referred to as breakbone fever, dandy fever, O'nyong-nyong fever, or seven-day fever [1]. In some cases, the disease progresses to more severe conditions like dengue hemorrhagic fever (DHF) and dengue shock syndrome (DSS), which can lead to bleeding, low platelet levels, and blood plasma leakage [3]. Furthermore, dengue infection can be complicated by hemophagocytic lymphohistiocytosis, which is rare and potentially life-threatening [4].

DHF typically affects children under the age of 15 years, although it may also occur in adults [5]. It is distinguished by a sudden onset of fever, which often lasts for two to seven days, as well as several nonspecific symptoms [3]. The revised classification (2008) divides dengue into two categories: non-severe and severe dengue (SDF); non-severe dengue is further divided into two categories: dengue with warning signs (D+W) and dengue without warning signs (D-W) [6].

The increase in international travel to regions where DF is prevalent has had a notable impact on the worldwide distribution of the disease, particularly given that many of these areas are popular tourist destinations [7]. Bali is recognized as one of the most popular destinations globally, and in this case, the patient developed symptoms after returning from Bali. Individuals who travel to regions where DF is prevalent are highly susceptible to contracting the disease and may also play a role in transmitting the disease to non-endemic regions [8]. Although Armenia is not considered an endemic area for DF, the recent discovery of *Aedes albopictus* in the country [9] is a cause for concern. This mosquito species is known to transmit the disease, and its presence in Armenia means that even a single imported case of dengue could have serious implications.

We present a case of a patient who returned from Bali and was admitted to the hospital with complaints of general weakness, temperature rise of up to 39-40°C, chills, tremors, and joint pains. The patient's condition suddenly worsened and she began experiencing vaginal bleeding, a petechial rash in the upper and lower extremities, and progressive leukopenia and thrombocytopenia. However, because of the unavailability of a dengue diagnostic kit in Armenia, a final diagnosis of DHF could not be made, despite the patient having mosquito bites on her body. Instead, she was diagnosed with a hemorrhagic fever of an unknown cause.

## Case presentation

A 23-year-old female patient was admitted to the infectious diseases department with complaints of general weakness, a temperature rise of up to 40°C, chills, tremors, and joint pains. The patient had no significant medical history. She reported feeling unwell for the past two days after returning from Bali. She spent two weeks there. She traveled to rural locations to engage in hobbies like surfing. She experienced a sudden spike in body temperature, reaching 40°C, along with chills, tremors, body aches, and profuse sweating two days after returning.

Upon admission, the patient presented with a moderate impairment in her overall clinical status. On physical examination, her blood pressure was 100/70 mmHg, pulse rate was 108 beats per minute, and oxygen saturation was 98%. The tongue was dry and swollen, and the mucosa of the oropharynx was hyperemic. Further examination revealed mosquito bites on the skin, but the abdomen was soft to touch and non-tender, and urination was normal. The skin blanch test was negative. The patient's kidney function was adequate, and she was conscious and able to respond. There were no signs of meningeal or focal symptoms. Her serological test values on admission were as follows: erythrocytes were 4.80 x 10^12/L, hemoglobin was 134 g/L, mean corpuscular volume (MCV) was 93.1 fL, mean corpuscular hemoglobin (MCH) was 27.9 pg, mean corpuscular hemoglobin concentration (MCHC) was 299 g/L, leukocytes were 2.78 x 10^9/L, neutrophils were 84.1%, eosinophils were 0.2%, basophils were 1.2%, lymphocytes were 11.3%, monocytes were 2.2%, and the thrombocyte count was 195 x 10^9/L. MCHC, MCH, leukocytes, and eosinophil counts were below the normal range, and neutrophils were higher than the normal value (Table 1).

**Table 1:** Hematological findings of the patient during the hospital stay MCV: mean corpuscular volume; MCH: mean corpuscular hemoglobin; MCHC: mean corpuscular hemoglobin concentration; HCT: hematocrit; ALP: alkaline phosphatase; GGT: gamma-glutamyl transferase; AST: aspartate aminotransferase; ALT: alanine transaminase.

Tests		1st day	4th day	6th day	8th day	12th day
Erythrocytes (*10^12^/լ)		4.80	4.06	4.18	4.27	5.20
Hemoglobin (g/L)		134	121	123	126	151
MCV (fL)		93.1	85.0	84.4	84.5	85.2
MCH (pg)		27.9	29.8	29.4	29.5	29.1
MCHC (g/L)		299	35.1	34.8	34.9	34.1
HCT (%)			34.5	35.3	36.1	44.3
Leukocyte (*10^9^/լ)		2.78	1.57	2.71	3.65	4.19
Neutrophils (%)		84.1	37.0	28.0	32.9	435
Eosinophils (%)		0.2	2.5	1.5	3.0	2.1
Basophils (%)		1.2	0.6	0.7	0.5	0.5
Lymphocyte (%)		11.3	47.8	56.1	49.9	43.2
Monocyte (%)		2.2	12.1	13.7	13.7	10.7
Thrombocyte (*10^9^/լ)		195	54	92	201	378
Creatinine (µmol/L)		73	43			
Albumin (g/L)			32.32	39.31		47.39
Total bilirubin (µmol/L)		5.5	5.0	5.4	6.4	
Unconjugated		3.1	3.9		2.9	2.6
Conjugated		2.4	1.2		2.5	3.8
ALP			107.6		104.7	112.6
GGT			207.7		259.8	183.3
AST (U/l)		31.3	472.9		130.6	42.0
ALT (U/l)		17.8	300.2		193.6	97.7
Glucose (mmol/l)			4.83			
Protein			60.2			

**Figure 1:**
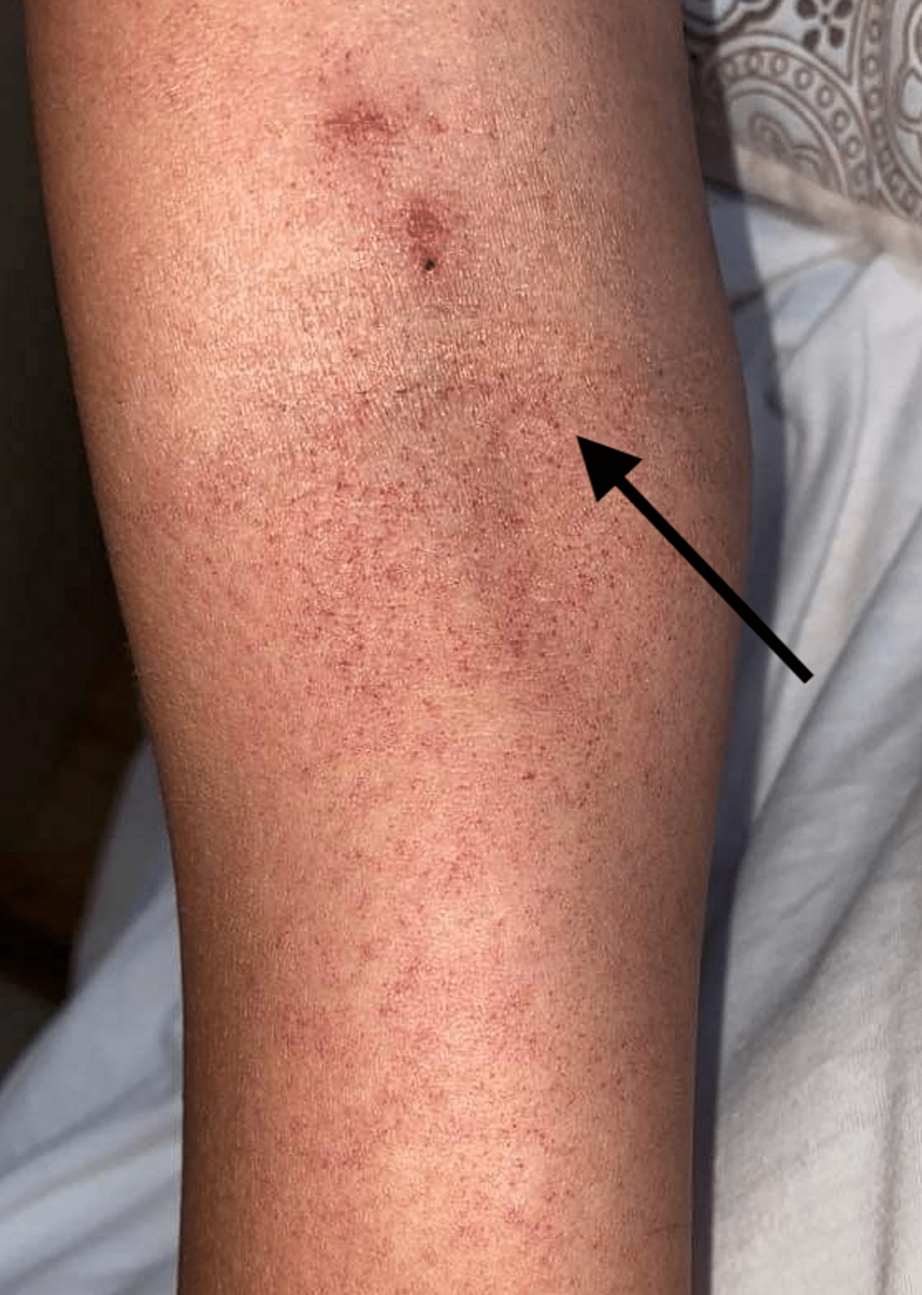
Small petechial rashes (black arrow) seen on extremities

On the fourth day of hospitalization, the patient's condition deteriorated. She started experiencing vaginal bleeding despite not menstruating at the time. The fever at this point was in the normal range. On the same day, the patient noted small petechiae rash sized 1-2 mm in diameter in the upper and lower extremities (Figure 1).

The patient's condition deteriorated, with progressive leukopenia (1.57 *10^9/L) and thrombocytopenia (54,000/mm3) (Table 1). Her serological test values changed significantly. A CT scan was done and splenomegaly was seen (spleen index: 720). Screening tests for HIV and hepatitis A, B, C, and E were performed, and the results showed that the patient was positive for anti-hepatitis C antibody. To confirm hepatitis C, a hepatitis C virus (HCV) polymerase chain reaction (PCR) was performed, but surprisingly it turned out to be negative. Finally, a consultation with a hematologist was done.

The physicians administered tranexamic acid (10 ml) for vaginal bleeding. Later, on consultation with a hematologist, Fraxiparine 0.3 ml s/c and frozen plasma were given. Despite the presence of mosquito bites, the final diagnosis of dengue fever could not be confirmed due to the unavailability of diagnostic kits. The patient was diagnosed with a hemorrhagic fever of unknown etiology with a severe clinical course.

The patient received infusion therapy, ceftriaxone 2 g (for seven days), frozen blood plasma (two units), and Fraxiparine 0.3 ml. Her condition improved, and upon discharge, she was in a state of recovery.

## Discussion

Dengue is a disease caused by the dengue virus, which is classified as an arbovirus. When an individual is infected with dengue, a range of clinical symptoms may present, which can vary from a mild form of the disease known as dengue fever (DF) to more severe and potentially life-threatening forms of the illness, such as DHF or DSS [10]. The revised classification (2008) divides dengue into two categories: non-severe and severe dengue; non-severe dengue is further divided into two categories: dengue with warning signs (D+W) and dengue without warning signs (D-W) [6]. The primary pathophysiological characteristic that differentiates DHF from DF is plasma leakage. The mechanism for pathogenesis is changes in the expression of adhesion molecules, enzymes, and cytokine receptors on endothelial cells, which are implicated in increasing the vascular permeability as well as activation of the coagulating system. The two fundamental pathological attributes of DHF are plasma leakage and intrinsic coagulopathy [11]. There are no specific medications to treat DF in any of its clinical manifestations [10]. Two types of dengue vaccines are available: QDENGA and DengVaxia. QDENGA is a live tetravalent attenuated vaccine for adults, adolescents, and kids from four years of age. DengVaxia, a tetravalent vaccine, was licensed to prevent severe secondary dengue in seropositive individuals [11].

In this report, we present the case of a young female patient who first visited the hospital with general weakness, temperature, chill, tremors, and some joint pain but subsequently developed a petechial rash, and started experiencing vaginal bleeding. The patient developed symptoms after returning from Bali. She spent two weeks there. She traveled to rural locations to engage in hobbies like surfing. Bali Province has been an endemic area to DF for years [12]. A study by Masyeni et al. confirms that dengue is one of the causes of fever in travelers visiting Bali [13]. Our patient just after returning from Bali showed symptoms and had mosquito bites on her body, which made a very high probability of dengue due to the nation's hyperendemic dengue illness and designation as a country with frequent or ongoing hazards of dengue infection for tourists on the dengue-risk map [14].

There are four main clinical indications of DHF, which include severe fever, bleeding, frequently accompanied by hepatomegaly, and in severe instances, circulatory failure [15]. Early stages of dengue infection can resemble the flu or other illnesses, including malaria, influenza, leptospirosis, yellow fever, Kyasanur Forest disease (KFD), viral hepatitis, chikungunya, or Zika [16]. Retro-orbital pain, fever, excruciating headache, excruciating joint and muscular pain, and nausea are symptoms of the illness [17]. When the patient was admitted initially, she was diagnosed with a hemorrhagic fever of unknown cause. Malaria was ruled out as pathogens for malaria were not detected in the thin blood smear and the thick drop. We were suspecting either chikungunya or dengue but after three days when the patient started showing hemorrhagic manifestations, progressive leukopenia, and thrombocytopenia, the suspected diagnosis became more inclined toward dengue. Dengue can result in more serious disease symptoms, like hemorrhage and subsequent vascular leaks, in addition to self-febrile sickness. Patients may exhibit pleural effusion, hemorrhage, thrombocytopenia with less than 100,000 platelets per liter, increased hematocrit levels, restlessness, nausea, and a sharp drop in body temperature during the disease's severe presentation [18]. In our case, the patient on the 4th day exhibited heavy vaginal bleeding, which is a symptom of DHF seen before. In a study by Malavige et al., vaginal bleeding was seen in 15.9% of total females, none of whom were menstruating at the time of infection [19].

During the evaluation of the patient, the healthcare team ordered tests for hepatitis A, B, C, and E viruses. Although the HCV antibody test returned positive, the subsequent HCV PCR test came back negative. This discrepancy led the team to investigate further and consider the possibility of cross-reactivity between the dengue virus and HCV, as they both belong to the *Flaviviridae* family. Cross-reactivity can occur in serological tests, causing false-positive results, and it is important to consider this possibility when interpreting results to avoid unnecessary treatments or misdiagnoses [20]. Therefore, in this case, the positive HCV antibody result could have been due to cross-reactivity with the dengue virus, highlighting the importance of careful interpretation of laboratory results in the diagnosis of infectious diseases.

Typically, the clinical course follows three phases: febrile, critical, and recovery. For our patient, the febrile phase was for three days when the patient had a high fever and other symptoms followed by the critical phase, which lasted about four days, when her condition deteriorated and she started having vaginal bleeding. The recovery phase was after seven days when along with treatment she started improving. Hematocrit decreased, while the white cell and platelet counts rose rapidly.

DF and DHF continue to be major global public health issues. Recent reports of DHF in various dengue outbreaks led to substantial fatality rates. There are currently no specialized antiviral medications that can be used as treatments or preventatives against dengue virus infection. Bed rest, antipyretics or tepid sponging to reduce the fever, analgesics or mild sedatives to aid with the pain, and fluid or electrolyte therapy to manage hydration are some of the recommendations for treating dengue [10]. This case report also highlights travel-related dengue, e.g., advising travelers to use insect repellent, a high index of suspicion in returned travelers from dengue endemic countries, and making testing available.

The infusion therapy and frozen blood plasma were given to our suspected dengue patient to help maintain hydration and prevent complications such as shock. Frozen blood plasma was given to replenish clotting factors since our patient had severe bleeding. The treatment was symptomatic.

In a recent study, it has been found that *Aedes albopictus* is present in Armenia [9], which along with probable dengue patients should be an alarming situation for the government of Armenia. Due to the unavailability of diagnostic kits for dengue in Armenia, the final diagnosis, in this case, could not be confirmed. The government of Armenia needs to invest in improving the availability of diagnostic kits for dengue and other viral hemorrhagic fevers to ensure timely diagnosis and treatment for patients returning from endemic areas.

## Conclusions

Hemorrhagic fevers are caused by a variety of viruses and can be life-threatening. Early diagnosis and appropriate treatment are crucial for a successful outcome. This case report underscores the importance of timely diagnosis and appropriate treatment of hemorrhagic fevers. The unavailability of dengue diagnostic kits in Armenia highlights the urgent need to invest in improving the availability of viral hemorrhagic fever diagnostic kits. Moreover, this case report emphasizes the importance of maintaining dengue surveillance in non-endemic nations and carefully evaluating and monitoring febrile patients who have returned from dengue-endemic nations.
